# Supplementary material for: Saccharomyces boulardii (CNCM I-745) improves intestinal damage in sepsis by remodeling intestinal flora structure
Source: Front Cell Infect Microbiol. 2025 Jul 31;15:1602792. doi: 10.3389/fcimb.2025.1602792 (PMC12350329; doi:10.3389/fcimb.2025.1602792)
Supplement: Supplementary file 1 [file DataSheet1.zip › summary/summary/7_Differential_analysis/2_LEfSe_result/Group_DW0_vs_BW0_0.05_3.0/LEfSe_Group_DW0_vs_BW0.cladogram.pdf]

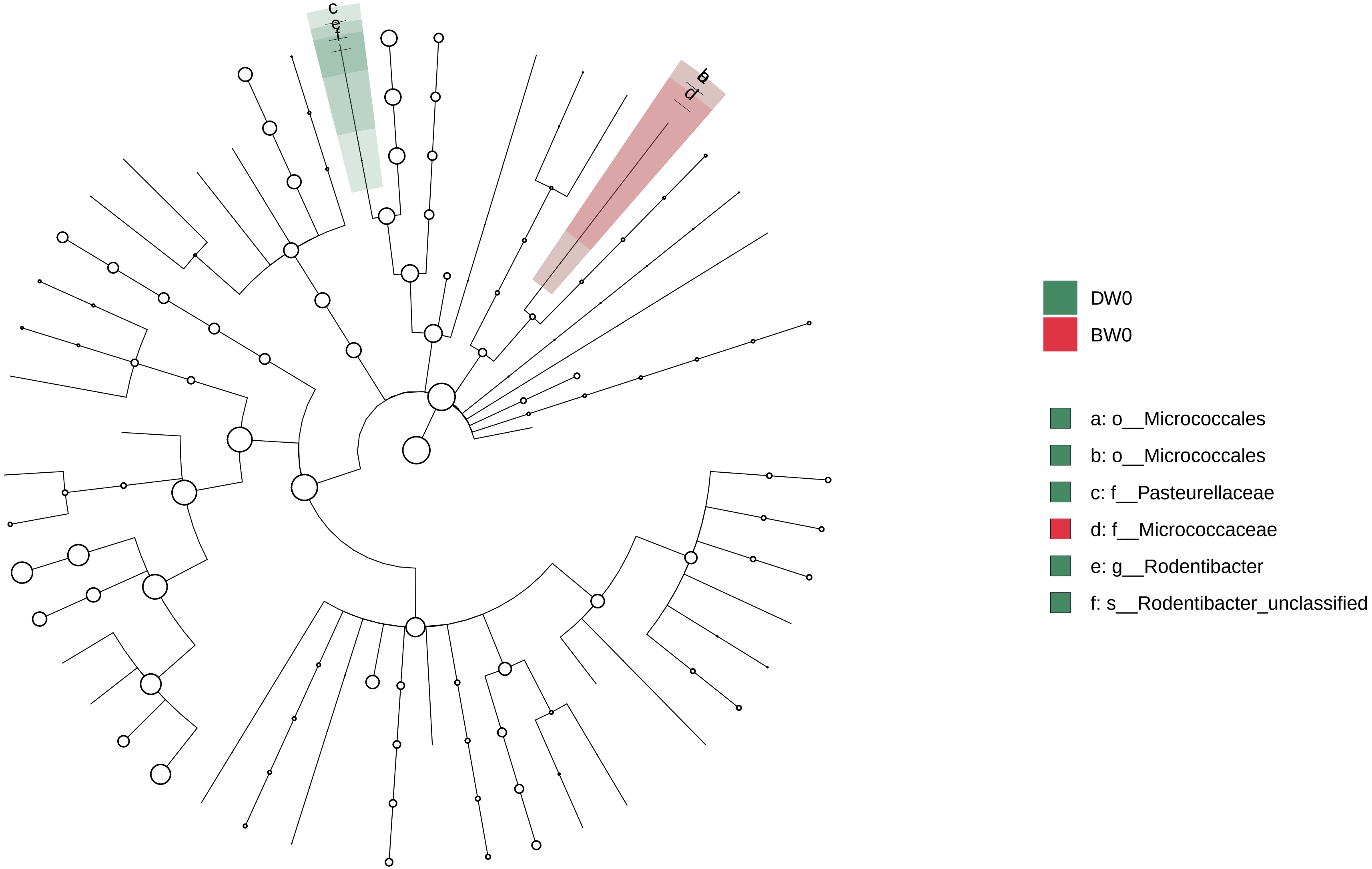

c
e
f
a
b
d
DW0
BW0
a: o__Micrococcales
b: o__Micrococcales
c: f__Pasteurellaceae
d: f__Micrococcaceae
e: g__Rodentibacter
f: s__Rodentibacter_unclassified
